# Experimental Investigation and Optimization of Electric Discharge Machining Process Parameters Using Grey-Fuzzy-Based Hybrid Techniques

**DOI:** 10.3390/ma14195820

**Published:** 2021-10-05

**Authors:** Ankit Sharma, Vidyapati Kumar, Atul Babbar, Vikas Dhawan, Ketan Kotecha, Chander Prakash

**Affiliations:** 1 Chitkara College of Applied Engineering, Chitkara University, Punjab 140401, India; ankit.sharma@chitkara.edu.in; 2 Department of Mechanical Engineering, Indian Institute of Technology, Kharagpur 721302, India; vidyapatikumar.me@gmail.com; 3 Mechanical Engineering Department, Shree Guru Gobind Singh Tricentenary University, Gurugram 122505, Haryana, India; atulbabbar123@gmail.com (A.B.); vikas251999@gmail.com (V.D.); 4 Symbiosis Centre for Applied Artificial Intelligence (SCAAI), Symbiosis International (Deemed University) (SIU), Pune 412115, India; 5 School of Mechanical Engineering, Lovely Professional University, Phagwara 110001, India

**Keywords:** grey theory, fuzzy logic, process parameter, response, optimization

## Abstract

Electrical discharge machining (EDM) has recently been shown to be one of the most successful unconventional machining methods for meeting the requirements of today’s manufacturing sector by producing complicated curved geometries in a broad variety of contemporary engineering materials. The machining efficiency of an EDM process during hexagonal hole formation on pearlitic Spheroidal Graphite (SG) iron 450/12 grade material was examined in this study utilizing peak current (I), pulse-on time (T_on_), and inter-electrode gap (IEG) as input parameters. The responses, on the other hand, were the material removal rate (MRR) and overcut. During the experimental trials, the peak current ranged from 32 to 44 A, the pulse-on duration ranged from 30–120 s, and the inter-electrode gap ranged from 0.011 to 0.014 mm. Grey relational analysis (GRA) was interwoven with a fuzzy logic method to optimize the multi-objective technique that was explored in this EDM process. The effect of changing EDM process parameter values on responses was further investigated and statistically analyzed. Additionally, a response graph and response table were produced to determine the best parametric setting based on the calculated grey-fuzzy reasoning grade (GFRG). Furthermore, predictor regression models for response characteristics and GFRG were constructed, and a confirmation test was performed using randomly chosen input parameters to validate the generated models.

## 1. Introduction

Electrical discharge machining (EDM) is a sophisticated, advanced machining method that utilizes a number of discrete sparks to remove the material from a workpiece and produce the finished component to the desired form [1,2]. During the EDM process, an identical replica of the tool shape is produced on the machined component [3,4]. It is especially well adapted to creating complicated form profiles on electrically conductive materials with poor machinability [5]. This operation is devoid of mechanical stress, chatter/burr production, and vibration issues, as the tool and the workpiece are not in direct touch. The abrasion resistance of the work material has little influence on its machining performance since the material is removed by melting via increased localized heat production. Due to the absence of cutting force, exceptionally deep narrow holes with a greater aspect ratio format can be machined, if necessary, with a minimal depreciation of the tool [6,7]. It may even create complex cavities in one operation. However, this operation has a number of disadvantages, including the creation of a recast layer, a poor material removal rate, a long machining time and associated expenses, limited flexibility, and the capacity to machine solely electrically conductive materials. 

There are several input factors that may be adjusted during an EDM process when cutting a certain material. Examining all of the EDM process parameters during an actual machining operation is challenging since the quantity of input parameters increases the experimental time and expenses proportionally. Various electrically controlled variables, such as peak current, cycle time, polarity, inter-electrode spacing, gap voltage, and so on, as well as non-electrically controlled factors such as electrode material, dielectric pressure, nature of dielectric, electrode rotation, and so on, have been observed to have a significant impact on the machining efficiency of an EDM operation. As a consequence, it is always desirable to operate the EDM machine by keeping the optimum value of the various input variables in order to fulfill the criteria of improved response outcomes. It would also result in a faster production rate due to the shorter machining time. However, previous research has revealed that the efficiency of the EDM operation’s machining is strongly impacted by three input variables: peak current (I), pulse-on time (T_on_), and inter-electrode gap (IEG). In this research, a hexagonal tool was used to machine the workpiece to truly comprehend the influence of input variables on the response. As a result, this study article employs a hybrid of two prominent approaches, namely, Grey Relational Analysis (GRA) and Fuzzy Logic, to explore the impact of input factors on output variables while machining tool steel (D3 grade). This method also assists in the finding of the optimum parametric setting for the EDM machining operation to achieve the best possible response values (outputs). The primary problems encountered during machining were that numerous machining operations and readings were required for a single set of parametric intermix of process variables to eliminate machining ambiguity.

Mandal et al. [8] utilized an artificial neural network (ANN) to simulate an EDM process. Later, non-dominating sorting genetic algorithm-II (NSGA-II) was used to improve the EDM parameters. Bharti et al. [9] optimized different input parameters of a die-sinking EDM operation utilizing the controlled elitist NSGA method. The considered process was also modelled using an ANN with a back-propagation method. For an EDM operation, Baraskar et al. [10] utilized the NSGA-II approach to find the optimum combinations of pulse-on time (T_on_), discharge current (I), and pulse-off time (T_off_) to improve surface roughness (SR) and material removal rate (MRR) responses. During EDM operation of D3 die steel, Shivakoti et al. [11] examined the effects of utilizing deionized water combined with salt as a dielectric medium on outcomes such as MRR, tool wear rate (TWR), radial overcut (ROC), and taper. Later, the Taguchi technique was applied to improve the EDM operation parameters that were taken into consideration. Aich and Banerjee [12] applied the weight-varying multi-objective simulated annealing technique to develop the corresponding Pareto-optimal front for the simultaneous optimization of MRR as well as SR in an EDM operation. Radhika et al. [13] considered peak current (I), pulse-on time (T_on_), and flushing pressure (P) as the input variables of an EDM process. A hybrid optimization technique consisting of ANN and genetic algorithm (GA) was later employed to reduce SR and TWR, and increase MRR. A Pareto-optimal front was also developed offering a set of non-dominated solutions. Tiwari et al. [14] deployed the GA method to simultaneously optimize MRR and SR during an EDM operation. The corresponding Pareto-optimal solutions were subsequently proposed. Mazarbhuiya et al. [15] carried out experimental runs on the basis of the Taguchi layout and used the GRA approach to determine the optimal discharge current, flushing pressure, polarity, and pulse-on duration for maximizing MRR and minimizing SR values during an EDM operation. Satpathy et al. [16] examined a metal matrix composite of AlSiC with input parameters such as peak current, pulse-on-time, duty cycle, gap voltage, and output characteristics such as MRR, TWR, diametral overcut (Z), and SR and optimized it using the combination of principal component analysis (PCA) and technique for order preference by similarity to ideal solution (TOPSIS) method (PCA-TOPSIS). Mohanty et al. [17] determined the best discharge current (I), pulse-on time (T_off_) and voltage (V) for having better results of MRR, TWR, SR and ROC. Singh et al. [18] optimized MRR using the NSGA-II method and TWR in an EDM operation while considering I, T_on_, T_off_, and V as the input variables. Gostimirovic et al. [19] calculated the energy efficiency of an EDM operation using a mathematical model with respect to MRR and SR responses. Later, a collection of optimum solutions was derived via evolutionary multi-objective optimization for discharge energy taking into account I and T_on_ as the input variables. Ramprabhu et al. [20] applied passing vehicle search (PVS) as a multi-objective optimization technique for optimizing various EDM process input variables. The performance of the adopted technique was also compared with that of other intelligent computing models. Based on the GRA technique, Tharian et al. [21] implemented a multi-objective optimization of MRR and SR during an EDM operation of Al7075 alloy. Huu et al. [22] suggested, as a solution, the multi-objective optimization based on ratio analysis (MOORA) methodology for having the best results of MRR, SR and TWR throughout the EDM operation of SKD61 die steel with low-frequency vibration. Analytic hierarchy process (AHP) was utilized to estimate relative weights of the considered responses. Kumar et al. [23] employed the GRA technique to evaluate the impact of input variables such as I, T_on_, and T_off_ on different surface roughness characteristics during EDM machining of D3 tool steel. Niamat et al. [24] attempted to examine the implications of I, T_on_, and T_off_ on MRR, SR, and TWR in an EDM operation by using response surface methodology (RSM)-based regression models. To ensure sustainability while optimizing the conflicting responses, multi-objective optimization was used as well. Kumar et al. [25] suggested teaching learning-based multi-objective optimization (TLBO) for optimizing MRR, SR, TWR, ROC, and circularity error during an EDM operation, with the results compared to existing metaheuristic algorithms. Pradhan [26] examined the machining behavior of AISI D2 tool steel using commercial grade EDM oil and optimized the MRR, TWR, and radial overcut using an RSM-based GRA method. Laxman et al. [27] machined the titanium-based super alloy using I, T_on_, and tool lift time as input parameters and MRR and TWR as output parameters, then improved the response characteristics using the Taguchi-Fuzzy method. Surekha et al. [28] used kerosene oil as a dielectric medium while machining EN-19 alloy steel and used the grey-fuzzy technique to optimize the output variables, MRR and TWR. Using GRA-PCA, Payal et al. [29] optimized MRR, TWR, and SR while machining Inconel 825. Prayogo et al. [30] investigated the machining properties of ST 42 steel, measuring MRR and overcut using transformator oil as a dielectric medium and optimizing the response characteristics using the Taguchi-GRA method. Rath [31] and Singh et al. [32] used the Grey-Taguchi technique to solve the multi-objective optimization problem for the optimization of output variables such as MRR, TWR, and SR. Sharma et al. [33] machined the Inconel and Nimonic alloys and used the fuzzy GRA method to optimize MRR, SR, electrode wear rate (EWR), and overcut. Bhaumik et al. [34] used the GRA method to optimize MRR, SR, ROC, and TWR when machining titanium alloy (grade 6). Belloufi et al. [35] used fuzzy logic to optimize MRR, TWR, wear rate (WR), SR, ROC, circularity (CIR), and cylindricity (CYL) during the machining of AISI 1095 steel utilizing Kerosene oil as a dielectric medium. In addition, a lot of research has been done in this area, using the grey approach [36] and hybrid nature-inspired algorithms [37,38] as multi-objective optimization tools, as well as the TOPSIS technique [39] for parameter selection.

It has been observed that the majority of research has focused on three input parameters: peak current, pulse-on time, and inter-electrode gap. However, there is limited research available in the machining of SG 450/12 iron material using Castrol SE 180 EDM fluid as a dielectric and machining a hexagonal hole. The above-cited evaluation of the existing literature reveals that quantitative optimization of EDM processes has already caught the attention of the research community, and several optimization techniques, such as GA, NSGA-II, simulated annealing, PVS, particle swarm optimization (PSO), TLBO, etc., have been applied in this direction. Those adopted algorithms have too many algorithmic parameters, which if not properly tuned, may increase the computational effort and result in local optimal solutions. Similarly, numerous decision-making techniques, such as VlseKriterijumska Optimizacija I Kompromisno Resenje (VIKOR), TOPSIS, GRA, AHP, MOORA, etc., have also been utilized to determine the most feasible parametric mixes for EDM processes. In this paper, an endeavor is described which focuses on the experimental examination of the effects of several process variables of an EDM operation on its responses (outputs) through interaction plots during machining pearlitic SG iron 450/12 grade material. In this EDM operation, I, T_on_, and IEG were considered the process variables, whereas the removal rate of a material and overcut were considered the output. Furthermore, the GRA technique in conjunction with fuzzy logic was used to determine the best parametric combination for the aforementioned operation. Consequently, the estimated grey-fuzzy reasoning grade (GFRG) results would aid the appropriate processing designers in determining the most suitable configuration of the EDM process variables and optimizing all competing performance metrics. Furthermore, the analysis of variance (ANOVA) method was also utilized to recognize the contributions of the EDM input variables in evaluating the machining performance. Finally, surface plots were established to assist process engineers in deciding the particular mix of input variables required to achieve the desired values of the properly considered responses. As a result, the operational effectiveness of EDM operations may be greatly enhanced by applying this multi-objective optimization technique. 

## 2. Materials and Methods

### 2.1. Workpiece and Tooling

**Figure 1:**
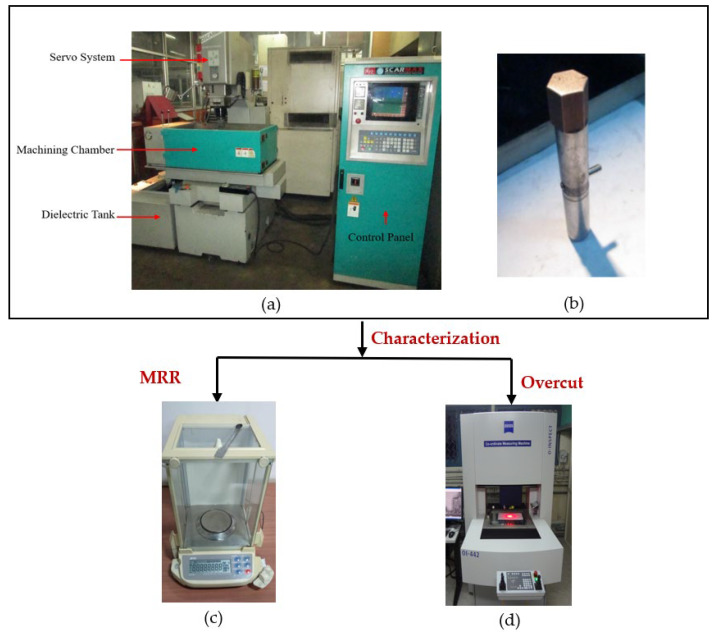
Electric discharge machining set-up. (**a**) Oscar electrical discharge machining machine. (**b**) Copper tool. (**c**) Weighing equipment. (**d**) Coordinate measuring machine (CMM).

In this research, the SG iron (grade-450/12, M/s Hindustan Malleables and Forge Ltd, Dhanbad, Jharkhand, India) material of the workpiece for EDM operation was chosen due to its several advantageous properties, such as good wear and corrosion resistance, better castability and machinability, reasonable strength, low cost, suitability for hydraulic applications as compared to steel, malleable and grey iron castings, capability to generate intricate shapes due to better fluidity as compared to steel castings, and requirement of less heat treatment resulting in better dimensional stability compared to malleable castings, hydraulic pump bodies, pump enclosures, pump casings, and pump hubs for diesel engine cooling systems. Table 1 and Table 2 indicate the chemical constitution of pearlitic ductile iron and mechanical properties of pearlitic SG iron (450/12 grade), respectively. In this research, a hexagonal-shaped copper tool was used for machining as shown in Figure 1b.

### 2.2. Experimentation and Characterization

Using an EDM set-up, experimental trials were carried out for producing hexagonal holes on pearlitic SG iron material (grade-450/12), with peak current, pulse-on duration, and inter-electrode spacing as adjustable process variables. The results of each of the variables under discussion were varied during the EDM operation on SG 450/12 iron material at four distinct operating levels, as implied in Table 3. Table 4 lists the technical characteristics of the Oscar Max EDM machine (OSCAR EDM Manufacturers, Taichung, Taiwan), and Figure 1a exhibits a picture of the EDM set-up, which was used to conduct sixteen experiments using a three-factor, four-level Taguchi orthogonal array. Voltage and pause time were kept constant at 45 V and 40 μs, respectively. Castrol SE 180 EDM fluid (Broughton lubricants, Preston, UK) was utilized as the dielectric fluid throughout the machining process because of its several benefits, including low smell, enhanced stability with prolonged life, low viscosity, high flash point, increased dependability, and safe usage. The size of the specimen was 15 × 40 mm^2^.

It is worth mentioning that the sixteen trial runs were done in a random order to minimize machining error, with the two most critical outputs, MRR and overcut, being considered as responses. The initial and final weights of the specimen were acquired using the weighing equipment (A&D GR-202, Tokyo, Japan) illustrated in Figure 1c, and the net difference in weight was divided by the product of material density and machining time to determine the MRR. Furthermore, as depicted in Figure 1d, overcut was assessed using a coordinate measuring machine (CMM) (ZEISS O-INSPECT 442, Jena, Germany) with GEOMET universal CMM software (Geomet Version 7, 2010, Helmel Engineering Products, Inc., Niagara Falls, NY, USA) with an accuracy of 4.5 μm.

### 2.3. Grey Relational Analysis(GRA)-Based Grey-Fuzzy Technique

Deng [40] developed the grey system idea, which refers to the rudimentary data in an impoverished, incomplete, and ambiguous system. The term “grey relation” refers to an inadequate connection of expertise inside a dataset. The GRA technique analyzes numerical data sequences to quantify the level of correspondence between the idealized and empirical levels (response values). The estimated level of sequence similarity is denoted by the grey relational coefficient (GRC). The GRC value will be one if two parameters of the elements under evaluation appear to be of equivalent significance. The GRA methodology may therefore be used to transform multiple-response variables to a single grey relational grade (GRG) value by taking into account mean GRC results for each dataset. Following that, the choice with the highest GRG result is deemed the most preferred option.

The Taguchi technique was used to estimate the most pertinent operating conditions for a certain expected quality. As a result, it was created with the express purpose of enhancing a particular quality attribute. It is obvious that the items must have a high-quality characteristic to satisfy the demands of the clients. The Taguchi approach involves the utilization of technical expertise to recognize the optimal input parameters for obtaining certain output values which could result in the vagueness of the decision-making procedure. The notion of grey system theory can effectively solve this flaw. This method reduces the number of stated quality criteria to a singular GRG value. The estimated GRG results are compared to satisfy the requirements of achieving the most preferred response results to evaluate the best possible operating levels of various input variables.

Several stages must be followed in order to conduct GRA, and these processes are outlined below:

Step 1: Normalize the experiment results.

In order to decrease variability and make the decision matrix dimensionless, the obtained results are first normalized to put them within a range from 0 to 1. The following normalization formula could be used based on the type of quality characteristics that are being put into consideration.

For beneficial characteristics:

(1) $$z_{p}^{*}\left(q\right)=\frac{z_{p}\left(q\right)-\min z_{p}\left(q\right)}{\max z_{p}\left(q\right)-\min z_{p}\left(q\right)}$$

For non-beneficial characteristics:

(2) $$z_{p}^{*}\left(q\right)=\frac{\max z_{p}\left(q\right)-z_{p}\left(q\right)}{\max z_{p}\left(q\right)-\min z_{p}\left(q\right)}\;p=1,2,...,m\;\mathrm{and}\;q=1,2,...,n$$

where $z_{p}\left(q\right)$ and $z_{p}^{*}\left(q\right)$ are the actual and normalized readings for *p*th choice with regards to *q*th criterion.

Step 2: Evaluation of GRC

From the normalized data, Equation (3) is used to compute the GRC values for each response. GRC values are used to indicate the relationship between the best and normalized values.

(3) $$\xi_{p}\left(q\right)=\frac{\delta_{\min}+\eta \delta_{\max}}{\delta_{0p}\left(q\right)+\eta \delta_{\max}}$$

where $\;\delta_{0i}\left(j\right)$ is the difference between the results, $z_{p}^{0}\left(q\right)$ (idealized sequence) and $z_{p}^{*}\left(q\right)$, and *η* is the distinctive characteristic with results ranging from 0 and 1 (*η* = 0.5 is frequently favored). It is primarily accountable for the extension or reduction of the range of GRC values. Additionally, $\delta_{\min}=\forall q^{\min}\in p\forall q^{\min}∥z_{0}\left(q\right)-z_{q}\left(q\right)∥$ is the minimum value of *δ*_0*p*_, and $\delta_{\max}=\forall q^{\max}\in p\forall q^{\max}∥z_{0}\left(q\right)-z_{q}\left(q\right)∥$ is the maximum value of *δ*_0*p*_. 

Step 3: Computation of GRG

Finally, for each of the options, the GRG results are generated using the average GRC values of the evaluated criterion.

(4) $$G_{p}=\frac{1}{n}\overset{n}{\underset{q=1}{\sum }}\xi_{p}\left(q\right)$$

The experimental run with the greatest GRG result is the preferred option delineating its dominance over others for a certain machining framework.

The fuzzy set theory [41] was mainly proposed to explain inaccuracies in the data in order to obtain a logical solution for every decision situation. In this research, the usage of beneficial and non-beneficial generates confusion, thus GRA employs fuzzy logic to address them.

A fuzzy set is made up of many membership functions that translate each component *p* into a universe of entities, such as P to a real number R in the (0,1) unit interval. The uncertainty of grey theory may be handled by establishing a fuzzy multi-performance tool utilizing a fuzzy logic method, often referred to as a grey-fuzzy logic approach. 

Fuzzifiers, membership functions, rule bases, inference engines, and defuzzifiers are all part of a fuzzy logic methodology. It requires fuzzifying the GRC data into linguistic words using membership functions that transform each input into some kind of membership value between zero and one for each input. After that, the inferential engine uses fuzzy logic to construct a fuzzy value based on the rule foundation. With the assistance of a defuzzifier, the resulting fuzzy value is converted to a binary value called the grey-fuzzy reasoning grade (GFRG). Fuzzy rules are used to relate the input grey relational coefficient to the output grey-fuzzy reasoning grade. The following is a typical representation of the set of rules:

First Rule: Fuzzy output (G is E1), if (w1 is A1) & (w2 is B1) & (w3 is C1) & (w4 is D1). Second Rule: Fuzzy output (G is E2), if (w1 is A2) & (w2 is B2) & (w3 is C2) & (w4 is D2). Nth Rule: Fuzzy output (G is En), if (w1 is An) & (w2 is Bn) & (w3 is Cn) & (w4 is Dn).(5)

where An, Bn, Cn, Dn and En are fuzzy elements which can be determined by the membership in the problem by the membership functions, i.e., µ_Ai_, µ_Bi_, µ_Ci_, µ_Di_ and µ_Ei_, independently under consideration. The fuzzy multi-response output, $\mu_{c_{0}}\left(G\right)$ may, thus, be enumerated utilizing the maximum-minimum interface approach. In a fuzzy system with a multi-response output and a variety of membership functions, the observational result may be expressed as follows:

(6) $$\mu_{G_{0}}\left(G\right)=\left(\mu_{A_{1}}\left(w_{1}\right)\wedge \mu_{B_{1}}\left(w_{2}\right)\wedge \mu_{C_{1}}\left(w_{3}\right)\wedge \mu_{D_{1}}\left(w_{4}\right)\wedge \mu_{E_{1}}\left(G\right)\right)\vee \;\left(\mu_{A_{2}}\left(w_{1}\right)\wedge \mu_{B_{2}}\left(w_{2}\right)\wedge \mu_{C_{2}}\left(w_{3}\right)\wedge \mu_{D_{2}}\left(w_{4}\right)\wedge \mu_{E_{2}}\left(G\right)\right)\vee \ldots \ldots \;\left(\mu_{A_{n}}\left(w_{1}\right)\wedge \mu_{B_{n}}\left(w_{2}\right)\wedge \mu_{C_{n}}\left(w_{3}\right)\wedge \mu_{D_{n}}\left(w_{4}\right)\wedge \mu_{E_{n}}\left(G\right)\right)$$

where symbol ‘˄’ denotes the minimization process and symbol ‘˅’ denotes the maximization process, respectively. Finally, several approaches, such as center of gravity fuzzification, weighted average, and mean of max membership, can be used to defuzzify the produced fuzzy output. The centroid or center of gravity fuzzification methodology is primarily utilized to transform the multi-response fuzzy output $\left(\mu_{c_{0}}\left(G\right)\right)$ into the equivalent binary value of GFRG, because it is the most prevalent and significant illustration of all techniques.

(7) $$\mathrm{GFRG}=\frac{\sum^{\;}{G\mu }_{G_{0}}\left(G\right)}{\sum^{\;}\mu_{G_{0}}\left(G\right)}$$

**Figure 2:**
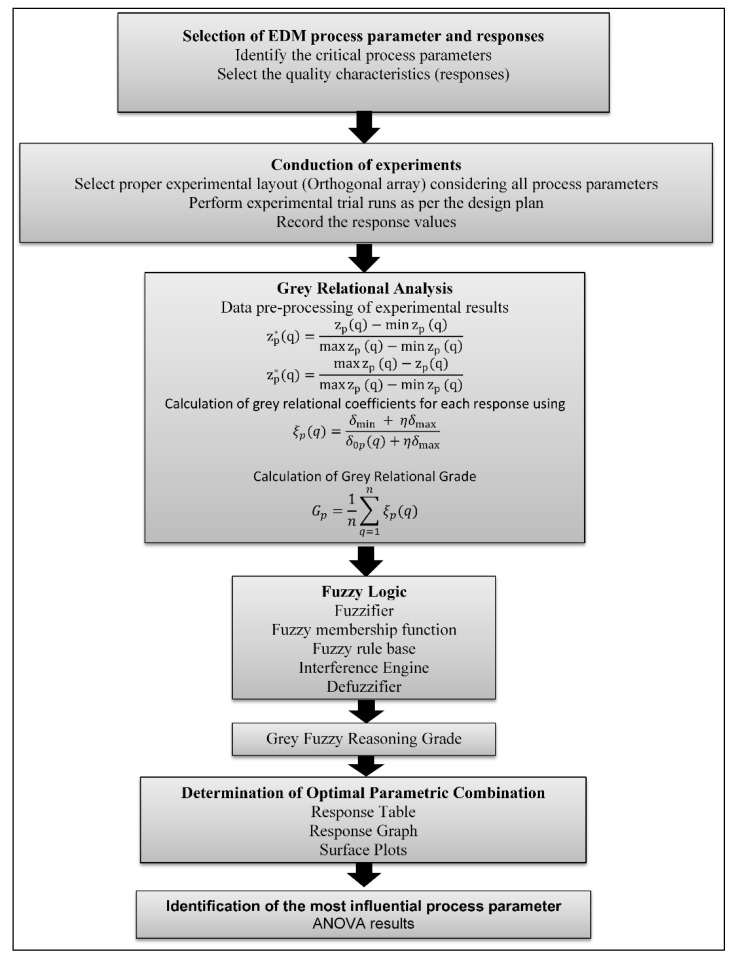
Grey-fuzzy approach for the EDM process.

The computed GFRG results could then be ranked in decreasing order, the preferred option being the alternative having the greatest GFRG value, which reduces unpredictability and ambiguity in the experimentally obtained data. The combination of the GRA methodology and fuzzy logic has evidently been a simple and effective way to solve multi-variable problems [42,43,44,45,46,47,48]. In this study, the grey-fuzzy method was utilized as a multi-criteria optimization procedure to evaluate the optimum intermix of input factors for the EDM process under consideration. Figure 2 shows the processes involved in using the grey-fuzzy method. The first and most important stage was to identify and choose key process and response parameters for the EDM operation, as indicated in Table 3. Once the parameters were defined, the appropriate experimental arrangement (Taguchi design) was determined while taking into account all process factors. The experimental trial was then carried out, and response values were assessed using different measuring equipment, as shown in Table 5. After obtaining the experimental layout shown in Table 5, grey relational analysis (GRA) was applied to that data, with the first step being data pre-processing using Equations (1) and (2), followed by a grey relational coefficient (GRC) evaluation using Equation (3), and finally a grey relational grade calculation using Equation (4). Following the application of GRA, the obtained result was coupled with the fuzzy logic technique and fed into the MATLAB environment (MATLAB 2013b, The MathWorks, Inc., Natick, MA, USA), where fuzzification and defuzzification of the membership function were performed using generated fuzzy rules based on the experimental data, and thus, the grey-fuzzy reasoning grade (GFRG) was calculated. Following that, a response table, response graph, and surface plots were generated in order to find the best parametric combination, and ANOVA was used to identify the significant parameters.

## 3. Results and Discussions

**Figure 3:**
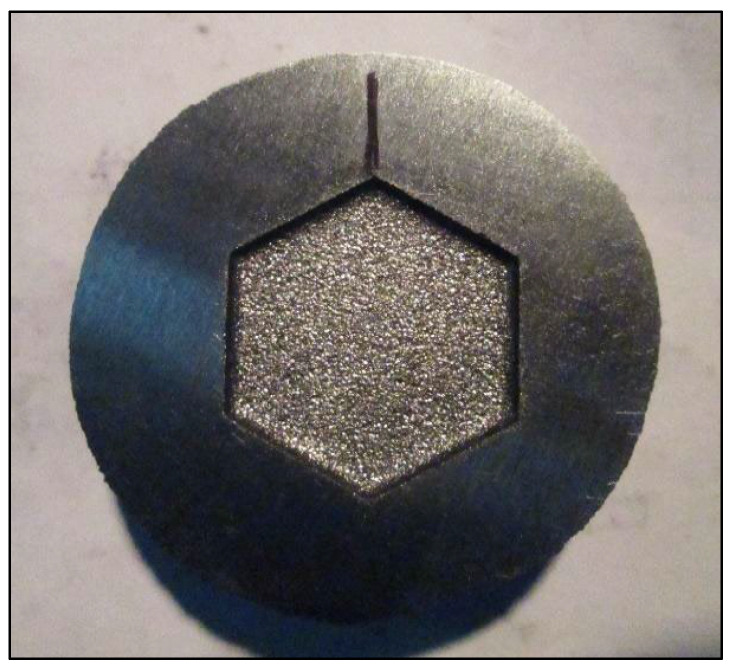
Machined workpiece.

The utilization of the proposed grey-fuzzy technique for determining the best possible parametric intermix for EDM machining of pearlitic iron material SG 450/12 is addressed in this section. Table 5 shows the experimental set-up as well as the measured results of the responses under consideration. The machined component (5 mm depth and 20 mm diameter) that was employed in the EDM operation is shown in Figure 3.

### 3.1. Effect of Input Variables on Material Removal Rate (MRR)

**Figure 4:**
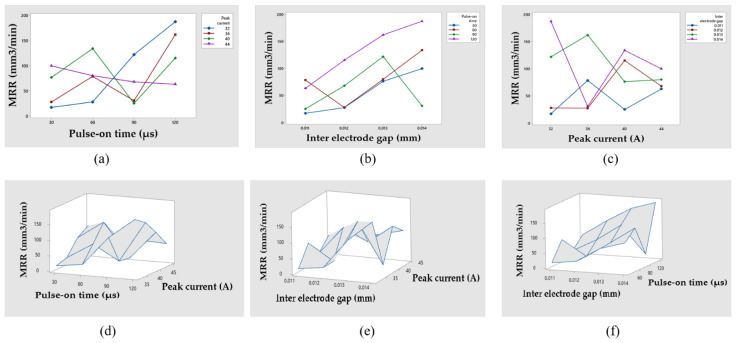
Influences of different EDM input variables on MRR with its surface plots. (**a**) Influence of T_on_ on MRR. (**b**) Influence of IEG on MRR. (**c**) Influence of I on MRR. (**d**) MRR vs. I and T_on_. (**e**) MRR vs. I and IEG. (**f**) MRR vs. T_on_ and IEG.

The removed volume of workpiece material divided by time is the material removal rate. MRR is greater when the pulse energy is higher [49]. Figure 4 shows the effects of several EDM input factors on MRR, as well as surface plots of MRR vs. input parameters. Figure 4a depicts the effect of T_on_ on MRR at various peak current levels. Figure 4b depicts the effect of IEG on MRR at various T_on_ levels. Figure 4c depicts the effect of I on MRR at various IEG levels. Figure 4d characterizes the surface plots of MRR vs. I and T_on_. Figure 4e illustrates the surface plots of MRR vs. I and IEG_._ Figure 4f demonstrates the surface plots of MRR vs. T_on_ and IEG.

The pulse-on time is the time period during which an electron discharge in the form of a spark occurs between the tool and the workpiece, causing more machining and material to be removed and, therefore, affecting the MRR. Since all the work is done during on-time, the duration of these pulses and the number of cycles per second (frequency) are important. Metal removal is directly proportional to the amount of energy applied during the on-time. In Figure 4a the increase in MRR with increasing T_on_ can be observed as the discharge channel created between the electrode and workpiece, which vaporizes more and more material from the workpiece. The available discharge energy increases as the T_on_ increases, resulting in greater melting and vaporization of the workpiece. The impulsive force in the spark gap rises as well, resulting in a larger MRR [25,49]. Figure 4d,f show a similar pattern of behavior.

In Figure 4b, it can be seen that when the IEG is increased, the MRR increases on different values of pulse-on time. The rise in IEG raises plasma resistance, which requires more energy to overcome [50]. This additional energy is supplied by the power source in the form of applied gap voltage and current. This electric energy is nothing more than the energy input that removes the material from its thermal impact. For a given gap voltage, increasing the gap distance increases plasma resistance, and increasing the gap distance increases discharge energy according to Joules Law (Q = I^2^RT) [50]. At the given gap distance, increasing the gap voltage reduces plasma resistance while continually increasing current until peak current is not reached. As a result of the strong thermal effect of electric discharge on the work material, material erosion rises with increasing IEG, which raises the discharge energy level [49,50]. Figure 4e,f illustrates that a similar effect can be inferred.

In Figure 4c, as the peak current is increased, the more spark energy is generated within IEG resulting in an increase in temperature, which can be seen at different values of IEG. When the temperature difference between the machining zones is raised, more material melts [51]. Furthermore, when the distance between the workpiece and the tool widens, the debris gathered during machining has more room to be flushed out of the machining zone, causing more material to be errored and ultimately leading to MRR [25], [51]. Figure 4d,e shows a similar pattern of behavior.

### 3.2. Effect of Input Variables on Overcut

**Figure 5:**
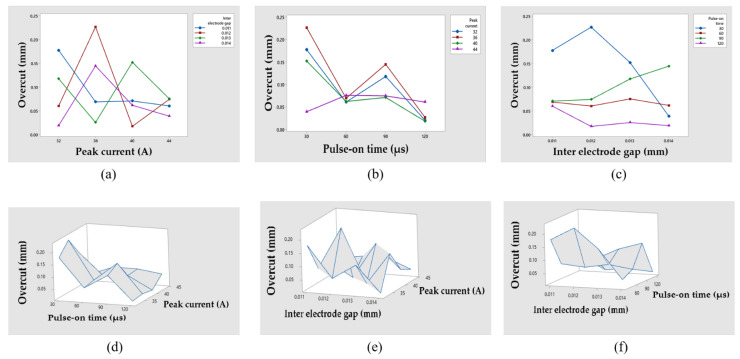
Effects of different EDM process parameters on Overcut with its surface plots. (**a**) Influence of T_on_ on Overcut. (**b**) Influence of IEG on Overcut. (**c**) Influence of I on Overcut. (**d**) Overcut vs. I and T_on_. (**e**) Overcut vs. I and IEG. (**f**) Overcut vs. T_on_ and IEG.

The discrepancy between the radius of the hole created and the radius of the electrode utilized is known as the overcut. Figure 5 shows the effects of several EDM input factors on overcut. Figure 5a depicts the effect of T_on_ on overcut at various peak current levels. Figure 5b illustrates the influence of IEG on overcut at various T_on_ levels. Figure 5c outlines the impact of I on overcut at various IEG levels. Figure 5d indicates the surface plots of overcut vs. I and T_on_. Figure 5e illustrates the surface plots of overcut vs. I and IEG_._ Figure 5f demonstrates the surface plots of Overcut vs. T_on_ and IEG.

Due to the existence of side sparks in the work material, overcutting is more common [51]. T_on_ is mainly responsible for overcut in Figure 5a. Due to a larger electric field, dielectric breakdown at a broad gap occurs at higher settings of these three EDM process parameters [52,53]. Increases in T_on_ are accompanied with an increase in overcut. Because the current flow between the machined component and the tool increases as T_on_ increases, this is expected. The more ions dissolve and the more hydrogen gas bubbles are created, the greater the current flow [52]. Figure 5d,f show a similar pattern of behavior.

Overcut rises when the IEG and T_on_ increase in Figure 5b. This is because overcut is mainly determined by the IEG and crater size [51]. When the IEG and T_on_ are both raised, bigger and wider craters develop, resulting in a greater overcut [51]. Furthermore, when the IEG increases, the gap widens and the time it takes for dissolution to complete increases, resulting in an increase in the overcut [52]. Moreover, for higher IEG values, spark density is greater, implying a quicker machining rate and, thus, side erosion is taken into account, resulting in a larger overcut [53]. Figure 5e,f illustrate that a similar effect can be inferred.

The overcut changes at various levels of IEG as the peak current rises in Figure 5c. This is owing to the fact that the distance between the tool and the workpiece changed as a result of IEG variation, and therefore, machining debris did not receive a consistent route to be flushed out of the machining zone, resulting in a variation in overcut. The overcut is primarily determined by peak current, and as peak current rises, so does the overcut [51]. As the peak current rises, a crater forms, resulting in the melting and vaporization of the machined region, resulting in the increment in overcut [52,53]. Figure 5d,e show a similar pattern of behavior.

### 3.3. Statistical Analysis

The ANOVA was used to discover which factors had the most influence on the MRR results. Furthermore, a regression equation was constructed that connected the process variable with the MRR values and defined the appropriate surface plots, as illustrated in Figure 4d–f. The results of an analysis of variance (ANOVA) based on the MRR values are shown in Table 6. A regression Equation (8) with a coefficient of determination (R^2^) of 71.81% and an adjusted coefficient of determination (R^2^-adj) of 29.53% was also constructed to demonstrate the relationships between different EDM process parameters and MRR values.

MRR = 484 − 24.9 A + 4.66 B − 31,374 C + 0.061 A × A + 0.0127 B × B − 2,720,875 C × C − 0.170 A × B + 2644 A × C + 75 B × C(8)

The ANOVA results based on the overcut values are shown in Table 7. A regression Equation (9) with a coefficient of determination (R^2^) of 82.05% and an adjusted coefficient of determination (R^2^-adj) of 55.13% was also constructed to demonstrate the relationships between different EDM process parameters and overcut values.

Overcut = −4.63 + 0.1276 A − 0.01605 B + 465 C − 0.000564 A × A + 0.000003 B × B − 6731 C × C + 0.000225 A × B − 8.18 A × C + 0.400 B × C(9)

### 3.4. Optimization Results

The GRC and GRG values are, hence, estimated depending upon the experimental data given in Table 5. Experimental results were pre-processed between zero and one in order to classify them in a normalized limit, based on the class of quality attribute to be assessed and whether the Equations (1) or (2) was to be used. The GRC and GRG values for each experimental sample were then calculated using these normalized data and Equations (3) and (4), as shown in Table 8. Experiment 4, which had the greatest GRG value, was the most effective. The fuzzy logic technique, on the other hand, was used to increase the quality of the resulted solution while reducing the ambiguity and vagueness in the experimental findings.

**Figure 6:**
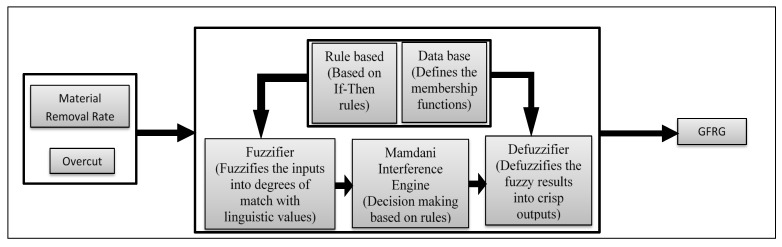
Two-in-one-out fuzzy Mamdani system.

**Figure 7:**
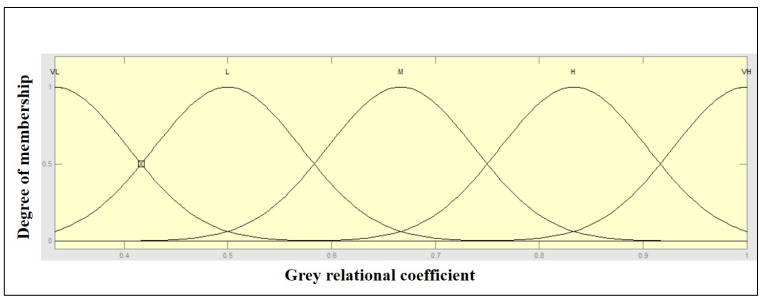
Input GRC Gaussian function with very low (VL), low (L), medium (M), high (H), and very high (VH).

**Figure 8:**
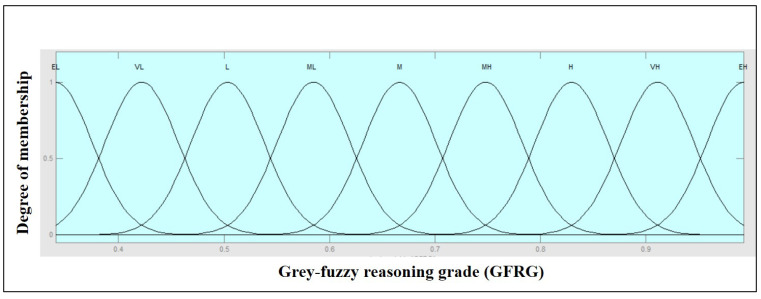
Output GFRG Gaussian function with extremely low (EL), very low (VL), low (L), medium low (ML), medium (M), medium high (MH), high (H), very high (VH), and extremely high (EH).

The MATLAB (2013a) fuzzy toolbox was used to generate output GFRG values in an analysis based on the grey-fuzzy method. The GRC values for the two outputs, i.e., MRR and overcut, were the entries to the fuzzy framework, whereas the GFRG was the output. In this way, the investigated multi-criteria problem could be modelled as a fuzzy two-in-one-out system, as shown in Figure 6. In Figure 6, the GRC value of the responses, such as MRR and overcut, was used as the input to the fuzzy logic. The collection of if-then rule bases was then created and sent into the fuzzifier, where the inputs were fuzzified into the degree of match with linguistic values. Furthermore, decision making was based on the Mamdani system, and the defuzzification of fuzzy outcomes into crisp output was performed, leading to the determination of GFRG. The input Gaussian membership function with five fuzzy subsets is shown in Figure 7. A Gaussian membership function with five fuzzy subsets was investigated in this context for input GRC values modified with minimum and peak values of 0.3333 and 1, as illustrated in Figure 7. Input fuzzy sets could be very low (VL), low (L), medium (M), high (H), and very high (VH). With nine subsets, Figure 8 shows the output membership functions. Another Gaussian membership function with nine fuzzy subsets was used to calculate the values of output GFRG, with minimum and maximum values ranging from 0.3404 to 0.9925. As demonstrated in Figure 8, output subsets of extremely low (EL), very low (VL), low (L), medium low (ML), medium (M), medium high (MH), high (H), very high (VH), and extremely high (EH) were all used.

Sixteen rules describing the relations among the GRC (input) and GFRG (output) results were developed based on sixteen sets of experimental trials. The following is an illustration of a rule such as this:

(GFRG is EL), if (MRR is VL) & (Overcut is VL).

**Figure 9:**
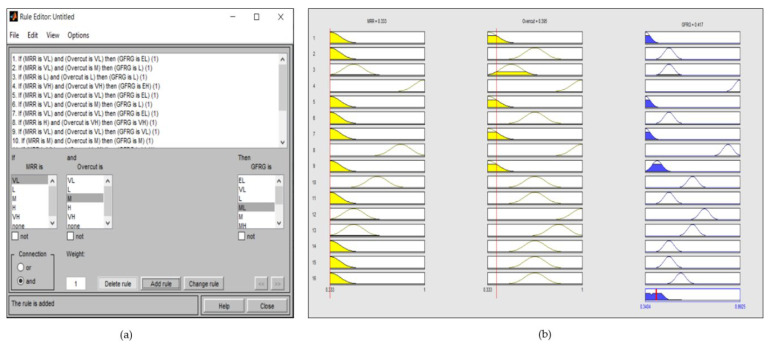
Pictorial illustration of elaborated rules. (**a**) Rule editor for the EDM process. (**b**) Rule viewer for the EDM process.

**Figure 10:**
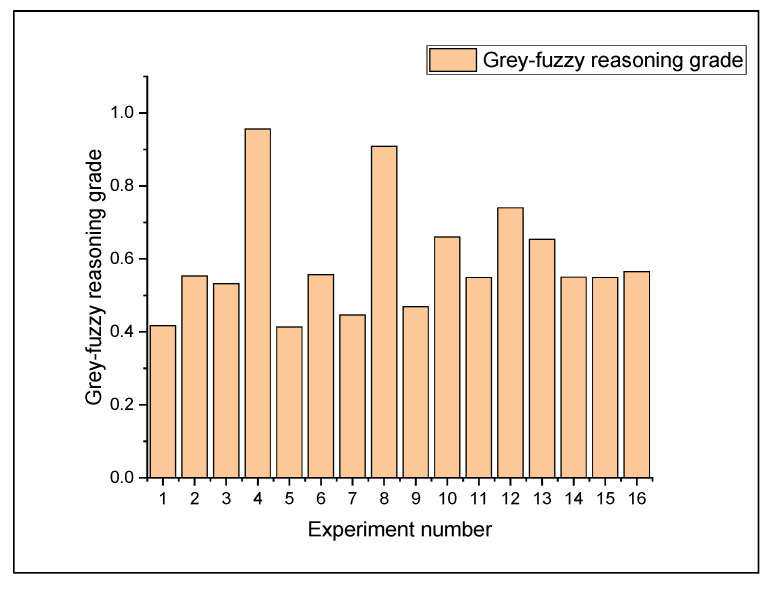
GFRG results for the experimental trails.

A pictorial illustration of these elaborated rules, obtained from the MATLAB tool box of the grey-fuzzy system, is shown in Figure 9a,b. The fuzzy rules generated depending on the sixteen groups of experimental runs are represented by the rows in Figure 9b, while the first two columns display the input GRC results for the two-response variable, and the final column provides the output GFRG value of the fuzzy system. The fuzzy rules generated from the sixteen sample sets are represented by the rows in Figure 9b, while the first two columns show the input GRC results for the two responses. On the other hand, the last column depicts the GFRG result of the fuzzy system. The height of the colored region for each Gaussian bell indicates the value of fuzzy associated with a fuzzy set’s membership function, and the position of each Gaussian bell shape in this picture denotes the fuzzy subset linked with the fuzzy rule. Following the application of grey-fuzzy, the GRC input value for MRR was 0.333, and the GRC input value for overcut was 0.395, as illustrated in Figure 9b. Furthermore, the initial experimental trial’s corresponding GFRG value was 0.417. Figure 10 also shows the GFRG results for all sixteen experiments. Using this number, it can be observed that the fourth experimental run, with the highest GFRG value of 0.9925, turned out to be the optimal parametric intermix for the EDM operation under consideration, which led to a simultaneous optimization of the response behavior.

**Figure 11:**
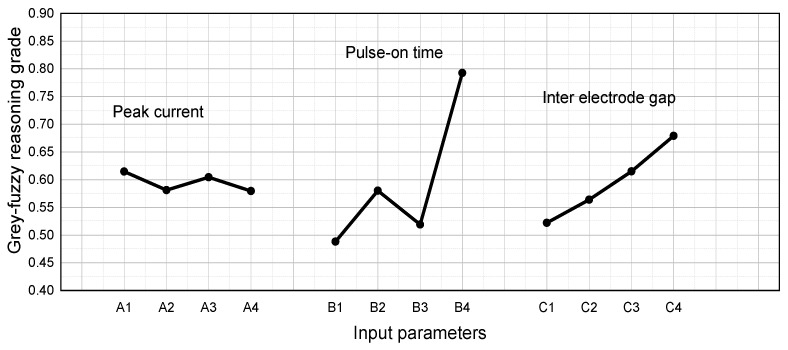
Response graph for GFRG values.

GFRG values, which were calculated by averaging the estimated GFRG value at different parametric conditions, are accentuated in Table 9, taking into account the mean of the calculated GFRG results at the respective operating levels of the input factors. The highest GFRG values for unique machining conditions are shown in bold. Figure 11 shows a response graph based on this table. Table 8 shows that the response values corresponding to the GFRG value for the current were greatest at the first operating level and lowest at the fourth operating level, as shown in Figure 11. The same type of fluctuation can be observed for the other two input variables, namely, pulse-on time and inter-electrode gap, which had the greatest GFRG values at the fourth operational level. The difference in slope for all input parameters demonstrates their importance, with pulse-on time having the steepest slope, as seen in Figure 11. The optimal set of machining parameters for EDM was found to be I = 32 A, T_on_ = 120 μs, and IEG = 0.014 mm, which can symbolically be denoted as A_1_B_4_C_4_. The max-min column in Table 9 reveals the T_on_ to be the most important process variable, which is further confirmed by its steep slope in Figure 11.

**Figure 12:**
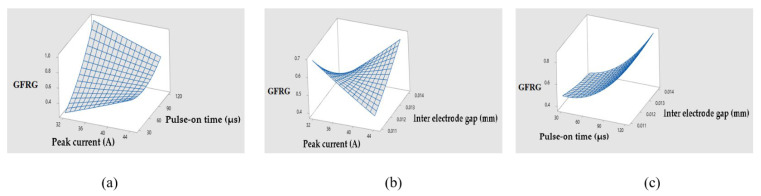
Surface plots of GFRG values w.r.t. process parameters. (**a**) GFRG vs. I, T_on_. (**b**) GFRG vs. I, IEG. (**c**) GFRG vs. T_on_, IEG.

The ANOVA results based on the GFRG values are shown in Table 10. A regression Equation (10) with a coefficient of determination (R^2^) of 82.91% and an adjusted coefficient of determination (R^2^-adj) of 57.27% was also constructed to demonstrate the relationships between different EDM process parameters and predicted GFRG values. Figure 12 depicts the surface plots produced from this regression equation, which generally illustrate the influence of numerous EDM process variables on the calculated GFRG values.

GFRG = 7.29 − 0.146 A + 0.0136 B – 739 C + 0.00013 A × A + 0.000050 B × B + …………….. + 5562 C × C − 0.000593 A × B + 14.54 A × C + 0.48 B × C(10)

As shown in Table 11, a validatory test was performed on the randomly selected input parameters, and Table 12 depicts a comparison of the findings acquired by previous researchers with the research examined in the current study. Using the established regression Equations (8)–(10), the corresponding values of the predicted values of MRR, overcut, and GFRG were computed. Furthermore, experimentation was carried out for the corresponding randomly chosen input parameters, and the difference between the predicted and experimental values was computed. It is clear that the difference between them was extremely minimal. According to Table 12, previous researchers used a variety of dielectric mediums throughout the machining process, including deionized water, kerosene oil, commercial grade EDM oil, and transformator oil on different workpiece materials. Our current investigation, as shown in Table 12, indicated that the MRR value was 187.005 mm^3^/min, whereas previous research work showed values around 18.619, 33.780, 10.339, 15.5844, 6.38141, 3.8370, and 59.95 mm^3^/min. This significant enhancement in material removal response characteristics was found as a result of a variety of parameters, including the use of a SG iron (pearlitic 450/12 grade) workpiece, Castrol SE 180 EDM oil as a dielectric medium, and various process parametric settings. Castrol SE 180 EDM oil is a low-viscosity, high-performance electric discharge machining (EDM) fluid that is specially designed and formulated to enable optimum rates of metal removal, as well as low electrode wear, good surface finish, and fine tolerances, and is equally suitable for roughing and finishing operations. Furthermore, the low viscosity guarantees that spark gaps are well cooled and flushed. Its excellent oxidation stability ensures a long service life. Overall, the aforementioned benefits result in an improved outcome in terms of response characteristics, particularly in 187.005 mm^3^/min of MRR and 0.0177 mm of overcut.

## 4. Conclusions

This study examined the parametric optimization and analysis of an EDM operation using pearlitic SG iron 450/12 grade material, with I, T_on_, and IEG as process factors and MRR and overcut as response variables. A grey-fuzzy logic technique was used to assess the optimum parametric intermix of those input process parameters. Based on the aforementioned findings and discussions, it is clear that the EDM process variables (input) must be maintained at I = 32 A, T_on_ = 120 s, and IEG = 0.014 mm in order to obtain the most suitable response values. The impact of different input factors on response characteristics was investigated. The ANOVA results also showed that none of the parameters were statistically significant. A response table and response graph were generated, from which it was deduced that pulse-on time was the most important of all process factors. Furthermore, validation tests for the developed regression equation were performed with randomly selected input parameters, and the error computed between the predicted and experimental values was found to be in the tolerance region, indicating that our developed regressions model is suitable for prediction.

As a consequence, it is possible to claim that a grey-fuzzy method based on a solid mathematical foundation may be successfully used to choose the optimum parametric mix for the EDM operation under discussion. Furthermore, the impact of more diverse responses, such as circularity error, tool wear rate, and surface roughness on the optimization performance of grey-fuzzy logic may be investigated. 

## Figures and Tables

**Table 1:** Chemical constitution of pearlitic ductile iron.

Element	C	Si	Mn	P	S	Cr	Mo	Cu	Mg	Ti	Zn	Fe	Others
%	3.365	2.393	0.238	0.072	<0.150	0.007	<0.010	0.37	0.085	0.032	0.027	90.75	2.661

**Table 2:** Mechanical properties of pearlitic SG iron (450/12 grade).

Mechanical Attributes.	Value
Ultimate tensile strength	450 × 10^6^ N/m^2^
Yield stress	310 × 10^6^ N/m^2^
Elongation	12%
Hardness	197 BHN
Volumetric mass density	6950 kg/m³
Relative wear resistance	Excellent

**Table 3:** Input factors and its operating levels.

Input Variables	Symbol	Unit	Operating Levels			
			1	2	3	4
Current (I)	A	A	32	36	40	44
Pulse-On Time (T_on_)	B	s	30 × 10^−6^	60 × 10^−6^	90 × 10^−6^	120 × 10^−6^
Inter-Electrode Gap (IEG)	C	m	11 × 10^−6^	12 × 10^−6^	13 × 10^−6^	14 × 10^−6^

**Table 4:** EDM set-up specifications.

Manufacturer	Model	Travel	Accuracy
Taiwan	Oscar-S 430	[X-400 Y-300 Z-300] mm	0.02 mm/300 mm

**Table 5:** Experimental details. Peak current (I). Pulse-on time (T_on_). Inter-electrode gap (IEG). Material removal rate (MRR). Overcut (OC).

Exp. No.	I (A)	T_on_ (µs)	IEG (mm)	MRR (mm^3^/min)	OC (mm)
1	32	30	0.011	17.125	0.1775
2	32	60	0.012	27.971	0.0605
3	32	90	0.013	121.719	0.1175
4	32	120	0.014	187.005	0.0193
5	36	30	0.012	27.546	0.2266
6	36	60	0.011	78.464	0.069
7	36	90	0.014	30.514	0.1445
8	36	120	0.013	161.831	0.0258
9	40	30	0.013	76.156	0.1522
10	40	60	0.014	133.575	0.0619
11	40	90	0.011	25.174	0.0709
12	40	120	0.012	115.08	0.0177
13	44	30	0.014	99.588	0.039
14	44	60	0.013	79.916	0.0756
15	44	90	0.012	67.694	0.0744
16	44	120	0.011	62.934	0.0605

**Table 6:** ANOVA results obtained for corresponding MRR values. Degree of freedom (DF). Sum of the Squares (SS). Mean of the Squares (MS).

Source	DF	Adj SS	Adj MS	F-Value	*p*-Value
Regression	9	28,461.1	3162.35	1.70	0.267
A	1	241.2	241.18	0.13	0.731
B	1	657.9	657.94	0.35	0.574
C	1	19.6	19.62	0.01	0.922
A × A	1	15.2	15.23	0.01	0.931
B × B	1	2075.7	2075.67	1.11	0.332
C × C	1	118.5	118.45	0.06	0.809
A × B	1	3673.0	3672.98	1.97	0.210
A × C	1	984.4	984.41	0.53	0.495
B × C	1	44.0	43.97	0.02	0.883
Error	6	11,171.4	1861.90	-	-
Total	15	39,632.5	-	-	-

**Table 7:** ANOVA results obtained for corresponding overcut values.

Source	DF	Adj SS	Adj MS	F-Value	*p*-Value
Regression	9	0.044724	0.004969	3.05	0.094
A	1	0.006323	0.006323	3.88	0.096
B	1	0.007804	0.007804	4.79	0.071
C	1	0.004303	0.004303	2.64	0.155
A × A	1	0.001301	0.001301	0.80	0.406
B × B	1	0.000123	0.000123	0.08	0.793
C × C	1	0.000725	0.000725	0.44	0.530
A × B	1	0.006400	0.006400	3.92	0.095
A × C	1	0.009418	0.009418	5.78	0.053
B × C	1	0.001270	0.001270	0.78	0.411
Error	6	0.009784	0.001631	-	-
Total	15	0.054508	-	-	-

**Table 8:** Normalized data, GRC and GRG values. Grey relational coefficient (GRC). Grey relational grade (GRG).

Exp. No.	Normalization Results		GRC		GRG
	MRR	Overcut	MRR	Overcut	
1	0	0.2350	0.3333	0.3953	0.3643
2	0.0638	0.7951	0.3482	0.7093	0.5287
3	0.6157	0.5223	0.5654	0.5114	0.5384
4	1	0.9923	1	0.9849	0.9925
5	0.0613	0	0.3475	0.3333	0.3404
6	0.3611	0.7544	0.4390	0.6706	0.5548
7	0.0788	0.3930	0.3518	0.4517	0.4017
8	0.8518	0.9612	0.7714	0.9280	0.8497
9	0.3475	0.3562	0.4338	0.4371	0.4355
10	0.6855	0.7884	0.6139	0.7027	0.6583
11	0.0474	0.7453	0.3442	0.6625	0.5034
12	0.5766	1	0.5415	1	0.7707
13	0.4854	0.8980	0.4928	0.8306	0.6617
14	0.3696	0.7228	0.4423	0.6434	0.5428
15	0.2977	0.7286	0.4159	0.6482	0.5320
16	0.2697	0.7951	0.4064	0.7093	0.5579

**Table 9:** Response table obtained for GFRG values. The highest GFRG values for unique machining conditions are shown in bold.

Input Variables	Operating Level				Max-Min	Ranking
	1	2	3	4		
Current	**0.6145**	0.5813	0.6045	0.5795	0.0350	3
Pulse-on time	0.4883	0.5800	0.5190	**0.7925**	0.3043	1
Inter-electrode gap	0.5220	0.5638	0.6150	**0.6790**	0.1570	2

**Table 10:** ANOVA results obtained for corresponding GFRG values.

Source	DF	Adj SS	Adj MS	F-Value	*p*-Value
Regression	9	0.310793	0.034533	3.23	0.083
A	1	0.008271	0.008271	0.77	0.413
B	1	0.005596	0.005596	0.52	0.496
C	1	0.010896	0.010896	1.02	0.351
A × A	1	0.000068	0.000068	0.01	0.939
B × B	1	0.033033	0.033033	3.09	0.129
C × C	1	0.000495	0.000495	0.05	0.837
A × B	1	0.044631	0.044631	4.18	0.087
A × C	1	0.029777	0.029777	2.79	0.146
B × C	1	0.001807	0.001807	0.17	0.695
Error	6	0.064074	0.010679	-	-
Total	15	0.374867	-	-	-

**Table 11:** Validation of regression equation.

Randomly Selected Input Parameters	Response	Predicted	Experimental	Error
A = 34 A, B = 50 µs, C = 0.011 mm	MRR (mm^3^/min)	39.4321	39.4100	0.0221
	Overcut (mm)	0.1051	0.1029	0.0022
	GFRG	0.5191	0.5184	0.0007
A = 38 A, B = 80 µs, C = 0.012 mm	MRR (mm^3^/min)	72.534	72.16	0.374
	Overcut (mm)	0.0882	0.0849	0.0033
	GFRG	0.5590	0.5579	0.0011
A = 42 A, B = 110 µs, C = 0.014 mm	MRR (mm^3^/min)	124.3180	123.926	0.392
	Overcut (mm)	0.0415	0.0402	0.0013
	GFRG	0.7815	0.7806	0.0009

**Table 12:** Results reported by past researchers and present study for different work material and dielectric fluid medium.

Authors	Work Material	Dielectric Fluid	Output Parameter	
			MRR (mm^3^/min)	Overcut (mm)
Mohanty et al. [17]	High Carbon Steel	Deionized Water, Kerosene oil	18.619	0.175
Pradhan M.K [26]	AISI D2 Tool steel	Commercial grade EDM Oil	33.780	0.005
Prayogo et al. [30]	ST 42 Steel	Transformator oil	10.339	0.087
Rath, U. [31]	EN19 alloy steel	EDM oil	15.5844	0.3
Sharma et al. [33]	Nimonic 90	EDM oil	6.38141	0.3022
Bhaumik et al. [34]	Grade 6 titanium alloy	EDM oil	3.8370	0.023
Belloufi et al. [35]	AISI 1095 steel	Kerosene oil	59.95	0.46
Present study	SG iron (pearlitic 450/12 grade)	Castrol SE 180 EDM	187.005	0.0177

## Data Availability

The data presented in this study are available on request from the corresponding author.
